# HIF-1α-Mediated, NADPH Oxidase-Derived ROS Contributes to Laryngeal Airway Hyperreactivity Induced by Intermittent Hypoxia in Rats

**DOI:** 10.3389/fphys.2020.575260

**Published:** 2020-10-07

**Authors:** You Shuei Lin, Yan-Jhih Shen, Ping-Hsun Ou, Ching Jung Lai

**Affiliations:** ^1^Department of Physiology, School of Medicine, College of Medicine, Taipei Medical University, Taipei, Taiwan; ^2^Ph.D. Program in Pharmacology and Toxicology, School of Medicine, Tzu Chi University, Hualien, Taiwan; ^3^Master Program in Medical Physiology, School of Medicine, Tzu Chi University, Hualien, Taiwan; ^4^Department of Physiology, School of Medicine, Tzu Chi University, Hualien, Taiwan

**Keywords:** capsaicin-sensitive superior laryngeal nerves, reactive oxygen species, hypoxia-inducible factor-1α, laryngeal airway hyperreactivity, intermittent hypoxia

## Abstract

Obstructive sleep apnea, similar to intermittent hypoxia (IH) during sleep, is associated with laryngeal airway hyperreactivity (LAH). IH-induced laryngeal oxidative stress may contribute to LAH, but the underlying mechanism remains unknown. Conscious rats were subjected to repetitive 75 s cycles of IH for 7 or 14 consecutive days. Reflex apneic responses to laryngeal provocations with chemical stimulants were measured to reflect laryngeal reflex reactivity. Compared with control rats, rats exposed to IH for 14 days, but not for 7 days, displayed enhanced apneic response to laryngeal chemical stimulants. The apneic response to chemical stimulants, but not to mechanical stimulation, was totally abolished by perineural capsaicin treatment of superior laryngeal nerves (SLNs) or by the sectioning of the SLNs, suggesting that the reflex was mediated through capsaicin-sensitive SLNs. Daily intraperitoneal administration of N-acetyl-L-cysteine [NAC, a reactive oxygen species (ROS) scavenger], apocynin (an inhibitor of NADPH oxidase) or YC-1 (an inhibitor of HIF-1α), but not their vehicles, largely attenuated this augmented apneic response in 14 days IH rats. Laryngeal lipid peroxidation (an index of oxidative stress) was elevated in 7 days IH rats and 14 days IH rats, and was abolished by any of these three pharmacologic interventions. The protein expression of HIF-1α (an index of HIF-1 activation) and p47^phox^ subunit in the membrane fraction (an index of NADPH oxidase activation) in the laryngeal tissues increased in 14 days IH rats; the former was reduced by NAC, whereas the latter was inhibited by YC-1. These results suggest that 14 days of IH exposure may sensitize capsaicin-sensitive SLNs and result in exaggerated apneic reflex response to laryngeal chemical stimulants. This phenomenon depends on the action of HIF-1α-mediated, NADPH oxidase-derived ROS.

## Introduction

Obstructive sleep apnea (OSA), characterized by intermittent hypoxia (IH) during sleep, is associated with laryngeal airway hyperreactivity (LAH) and laryngeal inflammation (Nandwani et al., 1998; Novakovic and MacKay, 2015). LAH is manifested by the increased sensitivity of laryngeal afferents to stimulants and further exaggerate several airway reflexes, such as cough (Hansson et al., 1992; Mutoh et al., 2000), apnea (Naida et al., 1996; Tsai et al., 2006; Liu et al., 2013), and laryngospasm (Mazzone and Canning, 2002). Among these laryngeal afferents, capsaicin-sensitive superior laryngeal nerves (SLNs), constituting a subpopulation of nociceptive-like free nerve endings, are highly sensitive to various inflammatory mediators (Tsai et al., 2009; Liu et al., 2013). Activation of capsaicin-sensitive SLNs may evoke a number of respiratory reflexes, such as apnea, cough, and glottis-stop reflex (Prudon et al., 2005; Liu et al., 2013), many of which are clinical signs of patients with OSA (Novakovic and MacKay, 2015; Gouveia et al., 2019). Also, patients with OSA exhibit chronic cough, a major symptom of LAH, which may be related to the degree of inflammatory markers in the airways (Chan et al., 2015). Several inflammatory mediators such as reactive oxygen species (ROS) can sensitize capsaicin-sensitive SLNs, leading to LAH development (Tsai et al., 2006; 2009). Furthermore, several inflammatory mediators have been demonstrated to elevate the excitability of sensory neurons in isolated nodose/jugular ganglionic origin of airway C fibers (Undem and Taylor-Clark, 2014), and thereby probably increases the sensitivities of capsaicin-sensitive SLNs. While the sensitization of capsaicin-sensitive SLNs is believed to be involved in the development of OSA-associated LAH, its underlying mechanisms have not been fully elucidated.

Obstructive sleep apnea-intermittent hypoxia is characterized by repetitive upper airway collapse during sleep and leads to repeated hypoxia/reoxygenation, resulting in ROS generation and increased oxidative stress (Lavie, 2003; Prabhakar, 2001). We previously reported that excessive ROS in the lungs of rats subjected to IH for 14 days is essential for sensitizing capsaicin-sensitive vagal afferents in the lower airways, and contributes to the development of the lower airway hypersensitivity (Yang et al., 2016). In that study (Yang et al., 2016), we also reported that IH-induced increase in ROS results from activation of NADPH oxidase by promoting the translocation of p47^phox^ subunit to the plasma membrane. In addition to ROS, IH activates hypoxia inducible factor-1 (HIF-1) (Yuan et al., 2005). HIF-1, a transcriptional activator, plays a vital role in regulating cellular and systemic oxygen homeostasis (Mole et al., 2009; Semenza, 2009). HIF-1 activation, as evidenced by stabilizing HIF-1α, can upregulate NADPH oxidase under IH challenge, which in turn enhances the ROS production (Yuan et al., 2011). Meanwhile, ROS generation is essential for increased HIF-1α expression in response to hypoxia (Bernard et al., 2018) or IH (Peng et al., 2006). HIF-1 activation (Dewitz et al., 2017; Sun et al., 2017) and ROS generation (Yang et al., 2016) each may independently promote airway inflammation. Thus, a positive interaction may exist between HIF-1 activation and ROS production following IH exposure; however, their involvement in IH-induced LAH remains to be explored.

We hypothesized that IH exposure may induce capsaicin-sensitive SLNs-mediated LAH in rats through HIF-1 activation and NADPH oxidase-derived ROS. In the present study, reflex apneic responses to laryngeal provocations with chemical stimulants were measured to reflect laryngeal reflex reactivity. IH was employed to induce LAH and the vital role of capsaicin-sensitive SLNs in this LAH was investigated. Pharmacological inhibitions of HIF-1α, NADPH oxidase, and ROS as well as associated biochemical analyses were performed to delineate their involvements.

## Materials and Methods

### Animals

Experiments were performed on male Sprague-Dawley rats (weight 320–420 g). All experimental procedures described below were approved by the Institutional Animal Care and Use Committee of Tzu Chi University.

### Exposure to IH

Rats were exposed to IH in a Plexiglas cylindrical chamber as described previously (Lai et al., 2006). In the chamber, oxygen concentration was gradually reduced from 20.9 to 5% by pure nitrogen infusion for 30 s with a timed solenoid valve and flow regulator. Then, compressed air was infused for 45 s to allow the gradual return of oxygen concentration to 20.9%. Rats were subjected to IH from 10:00 to 16:00 per day for 7 or 14 consecutive days. A similar pattern of gas dynamics in the chamber was followed for control room air (RA) animals, but pure nitrogen was replaced with compressed air. After the daily exposure period, the rats were placed individually in clear acrylic chambers.

### Animal Preparation

At 16 h after the last exposure to RA or IH, the rats were anesthetized by intraperitoneal injection of α-chloralose (100 mg/kg; Sigma Chemical, St. Louis, MO, United States) and urethane (500 mg/kg; Sigma) dissolved in a borax solution (2%; Sigma). The right femoral artery and left femoral vein were cannulated for the measurement of arterial blood pressure and intravenous administration of anesthetics, respectively. During the course of the experiments, supplemental doses of α-chloralose (20 mg/kg/hr) and urethane (100 mg/kg/hr) were administered to maintain the abolition of pain reflexes, which were induced by pinching the animal’s tail. The animal was tethered in a supine position, the neck was opened at the midline, and the bilateral SLNs were isolated carefully for the subsequent experiments. The body temperatures of the animals were maintained at ∼36°C throughout the experiment by using a servo-controlled heating blanket.

### Preparation of Functionally Isolated Larynx

The methods for preparing a functionally isolated larynx have been described in detail in previous studies (Lin and Kou, 1997; Tsai et al., 2006). In brief, a lower tracheal catheter (PE-260) was inserted caudally above the thoracic inlet, and an upper tracheal catheter (PE-200) was inserted cranially with its tip placed slightly below the cricoid cartilage.

### Laryngeal Provocations With Chemical Stimulants and Measurements of Reflex Responses

Laryngeal provocations with capsaicin, phenylbiguanide (PBG), α,β-methylene-ATP (α,β-meATP), and saline control were performed by administering the above stimulants into the laryngeal segment with a spinal needle for the assessment of IH-induced laryngeal reflex reactivity (Liu et al., 2013). Possible tachyphylaxis was prevented by allowing at least 20 min to elapse between two chemical challenges. Rats breathed spontaneously via the lower tracheal cannula. Respiratory flow was measured using a pneumotachograph (Fleisch 4/0; Richmond, VA, United States) coupled with a differential pressure transducer (Validyne MP45-12) and integrated to provide tidal volume (*V*_T_). Respiratory frequency, expiratory duration (*T*_E_), and *V*_T_ were measured on a breath-by-breath basis.

### Perineural Capsaicin Treatment of the SLNs

Bilateral SLNs were subjected to perineural capsaicin treatment as previously described for the selective blocking of the neural conduction of capsaicin-sensitive afferents (Liu et al., 2013). In brief, a segment (approximately 2 mm) of each SLN was wrapped in a cotton strip presoaked in capsaicin solution (capsaicin treatment; 250 μg/mL). After 15 min, the apneic reflex response to laryngeal capsaicin was abolished, and the cotton strips were removed. The blocking effect of capsaicin-sensitive afferent conduction by perineural capsaicin treatment was further confirmed by the absence of apneic reflex response to laryngeal capsaicin, whereas the presence of apneic reflex response to laryngeal mechanical stimulation. To perform laryngeal mechanical stimulation, the laryngeal mucosa was gently probed for 5 s by a nylon thread (0.3 mm diameter) to stimulate laryngeal afferents (Boushey et al., 1974; Liu et al., 2013).

### Measurement of Lipid Peroxidation in Laryngeal Tissues

After the ventilatory responses were measured, the laryngeal tissues were collected from sacrificed animals and frozen at −80°C. The levels of lipid peroxidation in the laryngeal tissues (∼50 mg) were measured using the concentrations of malondialdehyde (MDA), a product of lipid peroxidation, and a thiobarbituric acid assay kit (Cayman, Ann Arbor, MI, United States).

### Measurement of HIF-1α and NADPH Oxidase Subunit p47^phox^ in the Larynx

Protein expression of HIF-1α in the larynx was measured in assessing HIF-1 activation. The p47phox in the membrane and cytosolic fractions of laryngeal tissues was used to evaluate NADPH oxidase activation through a previously described method (Yang et al., 2016). Larynx homogenates were separated on 10% SDS-PAGE and transferred to polyvinylidene fluoride membranes (Merck Millipore Corporation, United States). After blocking with 5% bovine serum albumin, the blots were incubated with rabbit monoclonal HIF-1α primary antibody (1:2000; Cat. sc-10790, Santa Cruz) or goat polyclonal p47^phox^ (1:2000; Cat. ab166930, Abcam) at 4°C overnight and incubated with goat anti-rabbit secondary antibody. The protein bands were detected using an enhanced chemiluminescence kit (GE Healthcare, United States). The signals were visualized by exposing the membranes to X-ray films (Kodak) and quantified using ImageJ analysis system (National Institutes of Health).

### Pharmacological Agents

Capsaicin (0.08 μM), PBG (4.5 μM), and α,β-meATP (0.05 μM; 30 μL volume) were applied on the larynx. The agents were introduced into the laryngeal segment with a spinal needle. Treatments with N-acetyl-L-cysteine (NAC, an antioxidant, 300 mg/kg/day), apocynin (an inhibitor of NADPH oxidase; 30 mg/kg/day), and 3-(5′-hydroxymethyl-2′-furyl)-1-benzyl indazole (YC-1; an inhibitor of HIF-1α; 2 mg/kg/day) were administered daily by intraperitoneal injection 10 min prior to IH exposure for 14 consecutive days. A stock solution of capsaicin (5 mg/ml) was dissolved in 10% Tween 80, 10% ethanol, and 80% saline. The working solutions of capsaicin (0.08 μM) were prepared daily by further dilution by saline before use. Apocynin and YC-1 were prepared by dissolving in 20% dimethyl sulfoxide and diluting in saline before use. All the chemicals were purchased from Sigma-Aldrich (St. Louis, MO, United States) except YC-1 (Tocris, Ellisville, MO, United States). The effective doses for these specific inhibitors were adopted from previous studies (Kuo et al., 2011; Wang et al., 2016; Yang et al., 2016).

### Experimental Design and Protocols

In this study, 90 male adult Sprague-Dawley rats were divided into eleven groups for four series of experiments. All groups consisted of 10 rats, except in groups 4 and 9–11 comprised five rats per group. Laryngeal provocations of three different chemical stimulants, including capsaicin, PBG, and α,β-meATP, were performed in an alternative order. An elapsed time of ∼20 min was allowed between any two provocations of chemical stimulants for the respiratory pattern to return to control levels and prevention of any accumulated effect. In study series 1 (groups 1–4), reflex apneic responses to laryngeal provocations of three chemical stimulants (e.g., capsaicin, PBG, and α,β-meATP) were studied in rats exposed to RA for 14 days (group 1), IH for 7 days (group 2), and IH for 14 days (group 3) to assess the time-dependent effect of IH. Subsequently, apnea responses to these chemical stimulants were measured and after denervation or perineural capsaicin treatment with SLNs. The results were used in assessing the role of the capsaicin-sensitive SLNs. The effectiveness of perineural capsaicin treatment was verified by absence of apneic response to laryngeal capsaicin; its selectivity was further confirmed by persistence of apneic response to laryngeal mechanical stimulation in 14 day IH rats (group 4). In study series 2 (groups 5–8), reflex apneic responses to laryngeal stimulants were assessed in 14 day IH rats that received daily intraperitoneal injection of NAC (an antioxidant; NAC + IH14; group 5), apocynin (an inhibitor of NADPH oxidase; apocynin + IH14; group 6), YC-1 (an inhibitor of HIF-1α; YC-1 + IH14; group 7) or their vehicles (Vehicle + IH14; group 8) 10 min prior to IH exposure for 14 consecutive days. The roles of ROS, NADPH oxidase, and HIF-1 activation in LAH development by IH can be elucidated. In study series 3 (groups 1–7), various laryngeal tissue samples were obtained from all groups of rats at the end of these experiments. Levels of lipid peroxidation, HIF-1α, and p47^phox^ were measured for the assessment of the effects of IH on oxidative stress, HIF-1 activation, and NADPH oxidase, respectively, in the larynx. Particularly, levels of lipid peroxidation were compared among all groups to assess the involvement of NADPH oxidase and HIF-1 activation on oxidative stress induced by IH. Protein expressions of HIF-1α were compared between Vehicle + IH14 rats and NAC + IH14 rats to investigate the role of ROS in evoking HIF-1 activation by IH. Protein levels of p47^phox^ were compared between Vehicle + IH14 rats and YC-1 + IH14 rats to assess the role of HIF-1α in activation of NADPH oxidase. In addition, the effectiveness of NAC, apocynin, and YC-1 was also confirmed under the present experimental conditions. The levels of lipid peroxidation were investigated in NAC + IH14 rats and apocynin + IH14 rats. The protein expression of HIF-1α was investigated in YC-1 + IH14 rats. In study series 4 (groups 9–11) the apneic responses to laryngeal chemical stimulants were investigated in RA rats-treated NAC (group 9), apocynin (group 10), or YC-1 (group 11) to check the possibility of impairing sensory neuron function due to these pharmacological administrations.

### Data Analysis and Statistics

Baseline *T*_E_ was continually analyzed on a breath-by-breath basis as the average value over the 10-breath period immediately before the laryngeal provocation of stimulants. The longest *T*_E_ occurring during the first 10 s after laryngeal stimulants was divided by the baseline *T*_E_ to yield the apneic ratio; this step was done to compare the apneic responses induced by different stimulants. In this study, a breath with *V*_T_ > 20% of baseline *V*_T_ (averaged over 10 breaths) and a biphasic inspiratory and expiratory phase was recognized as effective. In all studies, baseline mean arterial blood pressure and heart rate were continuously analyzed at 1 s intervals and calculated as the mean value over the 10 s period immediately preceding laryngeal provocations. All physiological parameters were analyzed using a computer-based data acquisition system (MP150, BIOPAC Systems Inc., Goleta, CA, United States) and Acqknowledge 4.1 (BIOPAC Systems) software. Data for three or more groups were compared by one-way ANOVA or two-way mixed factorial ANOVA, followed by Neuman-Keuls test when appropriate. *p* < 0.05 was considered significant. All data are presented as mean ± SE.

## Results

### Baseline Physiological Parameters

After 14 days of IH or RA exposure, the difference in average body weight was not significant among all the groups as follows: RA rats (380.5 ± 10.3 g), 7 days IH rats (371.8 ± 7.3 g), 14 days IH rats (370.5 ± 8.9 g), 14 days IH rats with NAC treatment (372.5 ± 8.6 g), apocynin (368.4 ± 9.8 g), YC-1 (370.5 ± 8.1 g), and vehicle (376.3 ± 9.6 g), RA rats with NAC treatment (369. 2 ± 6.3 g), apocynin (373.9 ± 8.1 g), and YC-1 (362.9 ± 9.4 g). When anesthetized, the average mean arterial blood pressure (133.5 ± 2.8 mmHg) but not the heart rate (334.5 ± 12.8 beats/min) of 14 days IH rats were significantly greater than those of the RA rats (mean arterial blood pressure: 111.6 ± 2.2 mmHg; heart rate: 329.6 ± 9.5 beats/min). Also, treatment with NAC (109.1 ± 1.9 mmHg), apocynin (111.7 ± 2.0 mmHg), or YC-1 (116.7 ± 1.4 mmHg) but not their vehicle (131.0 ± 2.0 mmHg) prevented the elevation of mean arterial blood pressure caused by 14 days of IH treatment. However, no difference in average baseline respiratory frequency, *T*_E_, and *V*_T_ was observed among the groups.

### IH Exposure Enhances the Apneic Responses to Laryngeal Provocations of Chemical Stimulants

**FIGURE 1:**
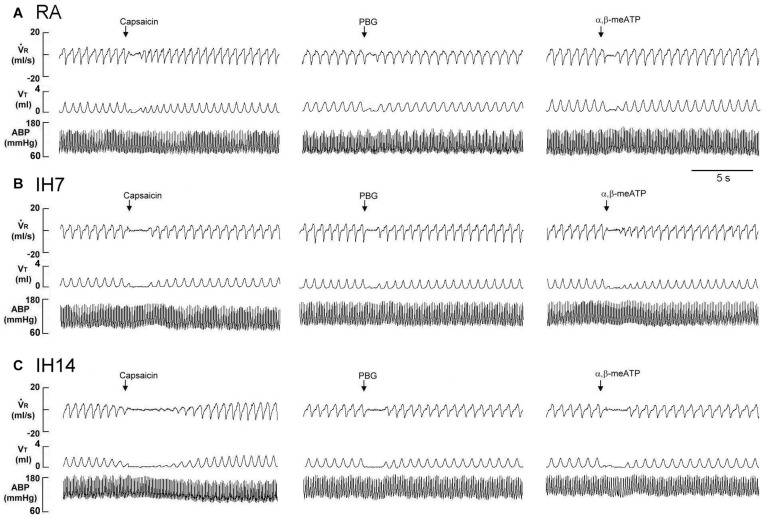
Immediate responses to laryngeal challenges of capsaicin, phenylbiguanide (PBG), and α,β-methylene-ATP (α,β-meATP) in three rats exposed to room air (RA) **(A)**, intermittent hypoxia for 7 days (IH7; **B**) or 14 days (IH14; **C**). Local application of capsaicin (0.08 μM), PBG (4.5 μM), and α,β-meATP (0.05 μM) (30 μL volume) were performed by careful installation of the chemical stimulants into the laryngeal segment via a spinal needle (indicated by an arrow). The elapsed time between the two challenges was 20 min. *V̇*_R_, respiratory flow; *V*_T_, tidal volume; ABP, arterial blood pressure.

**FIGURE 2:**
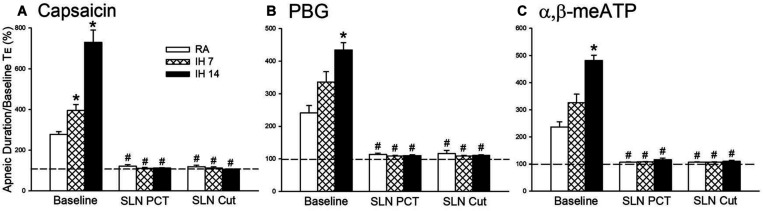
Mean apneic responses to laryngeal provocation of capsaicin **(A)**, phenylbiguanide (PBG; **B**), and α,β-methylene-ATP (α,β-meATP; **C**) from three study groups after exposure to room air (RA), intermittent hypoxia (IH) for 7 (IH7), or 14 days (IH14). The responses to provocation of each stimulant were measured before, during perineural capsaicin treatment (PCT) of superior laryngeal nerves (SLNs), and after bilateral section of SLNs (SLNs Cut). Capsaicin, PBG, and α,β-meATP were locally applied to the larynx. Apneic responses are reflected using the apneic ratio, which is defined as the longest expiratory duration (*T*E) occurring during the first 10 breaths after the provocation divided by the baseline *T*E. Horizontal dashed lines indicate an apneic ratio of 1 (100%; no response). *p < 0.05 compared with RA; #*p* < 0.05 compared with baseline responses in the same group. Data in each group are the mean ± SE from 10 rats. See the legend in Figure 1 for further explanation.

**FIGURE 3:**
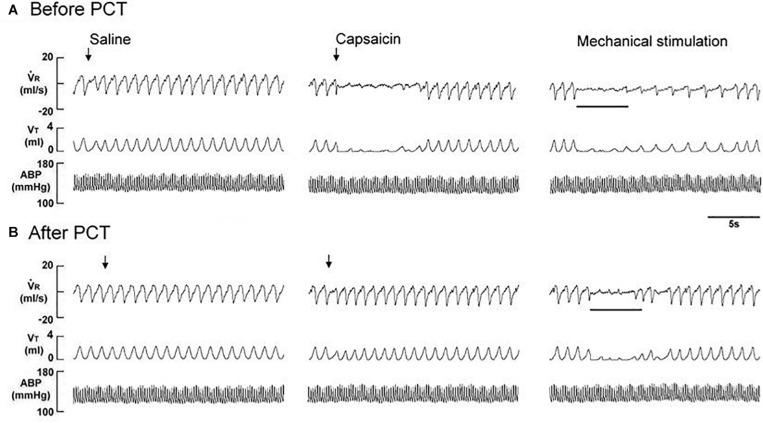
Immediate responses to laryngeal provocations of saline and capsaicin, and to laryngeal mechanical stimulation before **(A)** and after perineural capsaicin treatment (PCT; **B**) of superior laryngeal nerves in a rat exposed to intermittent hypoxia for 14 days. Saline or capsaicin (0.08 μM) in 30 μL volume was locally applied to the larynx (indicated by an arrow). Duration of laryngeal mechanical stimulation is indicated by the horizontal bars. The elapsed time between the two challenges was 20 min. *V̇*_R_, respiratory flow; *V*_T_, tidal volume; ABP, arterial blood pressure.

As shown in Figure 1, the laryngeal provocation of capsaicin in anesthetized spontaneously breathing RA rats induced a mild inhibitory effect on breathing and resulted in apneic response as evidenced by the prolonged *T*_E_. This apneic response resulted in an increase in apneic ratio, reflecting the magnitude of apneic response. The apneic response to the same dose of capsaicin was remarkably prolonged in 14 days IH rats compared with the apneic response of RA rats (Figures 1, 2A). In contrast, laryngeal provocation of saline failed to induce apneic response in 14-days IH rats (Figure 3). This potentiating effect of IH was not limited to the response elicited by laryngeal capsaicin. Similarly, the apneic ratio to laryngeal provocation of PBG (Figures 1, 2B) or α,β-meATP (Figures 1, 2C) was also significantly greater in 14 days IH rats than in RA rats. Compared with RA rats, the 7 days IH rats exhibited a small but significant increase in the apneic response elicited by laryngeal capsaicin (Figures 1, 2A); however, the apneic response to laryngeal provocation with PBG (Figure 2B) or α,β-meATP (Figure 2C) was not significantly enhanced.

### Role of Capsaicin-Sensitive SLNs in Apneic Reflex Responses to Laryngeal Chemical Stimulants

The perineural capsaicin treatment of SLNs did not cause any significant change in baseline respiratory pattern, but it nearly abolished the apneic reflex responses to laryngeal capsaicin (Figure 2A), PBG (Figure 2B), and α,β-meATP (Figure 2C) in RA rats, 7 days IH rats, and 14 days IH rats. In contrast, perinerual capsaicin treatment of SLNs failed to significantly alter the apneic response to mechanical stimulation in 14 days IH rats (Figure 3). The apneic ratios induced by laryngeal mechanical stimulation before and after perineural capsaicin treatment were 787.8 ± 25.9 and 748.5 ± 42.7%, respectively (*n* = 5, *p* = 0.462). Furthermore, the apneic reflex responses to the laryngeal capsaicin (Figure 2A), PBG (Figure 2B), and α,β-meATP (Figure 2C) were completely blocked by bilateral SLN denervation in those rats.

### Role of ROS, NADPH Oxidase, and HIF-1α in IH-Enhanced Reflex Apnea to Laryngeal Provocations of Chemical Stimulants

**FIGURE 4:**
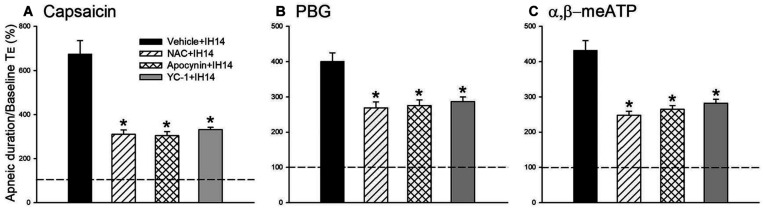
Mean apneic responses to laryngeal provocation of capsaicin **(A)**, PBG **(B)**, and α,β-meATP **(C)** from four groups after exposure to IH for 14 days with daily intraperitoneal treatment with N-acetyl-L-cysteine (NAC, an antioxidant; NAC + IH14), apocynin (an inhibitor of NADPH oxidase; Apocynin + IH14), YC-1 (an inhibitor of HIF-1α; YC-1 + IH14) or their vehicle (Vehicle + IH14). Apneic responses are reflected by the apneic ratio, as explained in the legend of Figure 2. Horizontal dashed lines indicate an apneic ratio of 1 (100%; no response). *p < 0.05 compared with Vehicle + IH14. Data in each group are the mean ± SE from 10 rats.

An antioxidant (NAC), an inhibitor of NADPH oxidase (apocynin), and an inhibitor of HIF-1α (YC-1) were used in assessing the suppressive effect on the potentiating effect of IH and investigating the roles of ROS, NADPH oxidase, and HIF-1 activation. Daily treatment with NAC or apocynin did not cause any significant change in the baseline respiratory frequency, *T*_E_, and *V*_T_ in 14 days IH rats compared with those of RA rats. The IH-enhanced apneic responses to capsaicin (Figure 4A), PBG (Figure 4B), and α,β-meATP (Figure 4C) were significantly attenuated by daily treatment with NAC or apocynin. Daily treatment with YC-1 also markedly reduced the potentiating effect of IH on apneic responses to laryngeal chemical stimulants (Figure 4). The suppressive effect of YC-1 treatment on IH-enhanced apneic responses to these chemical stimulants was similar to that produced by treatment with NAC or apocynin. However, their vehicles did not possess such a suppressive effect (Figure 4). The apneic ratios induced by capsaicin in 14 days IH rats and Vehicle + IH14 rats were 729.8 ± 60.4 and 673.6 ± 61.7% (*P* > 0.05), respectively. In addition, the apneic ratio evoked by PBG and α,β-meATP in 14 days IH rats (PBG: 427.0 ± 22.2%; α,β-meATP: 467.5 ± 19.0%) was similar those of Vehicle + IH14 rats (PBG: 398.7 ± 24.8%; α,β-meATP: 431.7 ± 27.8%).

### Laryngeal Levels of Lipid Peroxidation and Protein Expressions HIF-1α and p47^phox^

**FIGURE 5:**
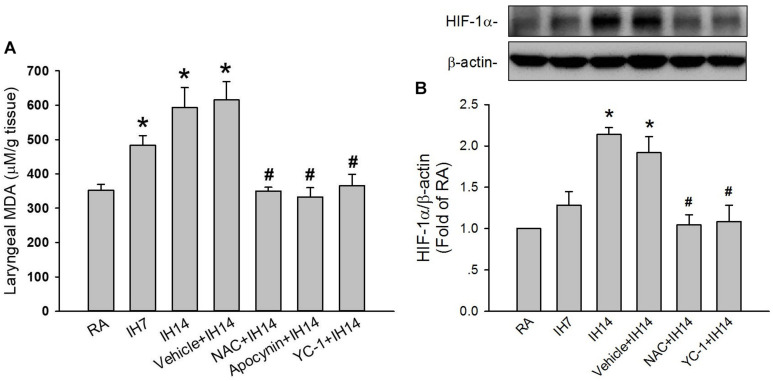
Level of malondialdehyde (MDA) and protein expression of HIF-1α in the laryngeal tissues of rats. **(A)** Three groups of rats were exposed to RA or intermittent hypoxia for 7 days (IH7) or 14 days (IH14), while the other four groups were exposed to IH14 with daily treatment with N-acetyl-L-cysteine (NAC + IH14), apocynin (apocynin + IH14), YC-1 (YC-1 + IH14) or their vehicle (Vehicle + IH14). The laryngeal levels of MDA, an indicator of oxidative stress, in seven groups were analyzed by an assay kit. **(B)** Three groups of rats were exposed to RA or IH for 7 days or 14 days, while the other three were exposed to IH14 with daily treatment with NAC (NAC + IH14), YC-1 (YC-1 + IH14) or their vehicle (Vehicle + IH14). The protein levels of HIF-1α in the laryngeal tissues were analyzed through Western blot analysis to assess HIF-1 activation. Data in each group are means ± SE of five rats. *p < 0.05 compared with RA; #*p* < 0.05 compared with Vehicle + IH14.

**FIGURE 6:**
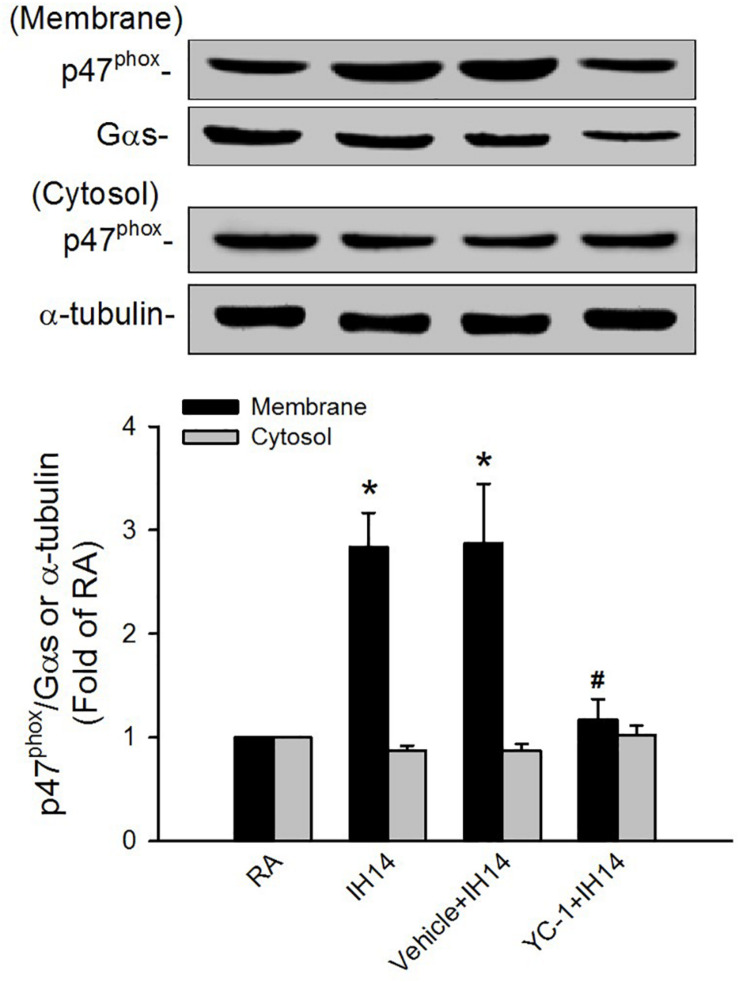
Protein expression of p47^phox^ in the laryngeal tissues of rats. Four groups of rats were exposed to room air (RA) or intermittent hypoxia (IH) for 14 days with or without daily treatment with YC-1 (YC-1 + IH14) or its vehicle (Vehicle + IH14). The protein expression levels of p47^phox^ in the membrane and cytosolic fractions of laryngeal tissues were measured by Western blot analysis to evaluate the activation of NADPH oxidase. Data in each group are means ± SE of five rats. *p < 0.05 compared with the level of the same fraction in the RA rats; #*p* < 0.05 compared with the level of the same fraction in the Vehicle + IH14 rats.

The lipid peroxidation level in the larynx was significantly increased in 7 or 14 days IH rats compared with that in RA rats (Figure 5A). Increases in lipid peroxidation in the laryngeal tissues in 14 days IH rats were completely prevented by daily treatment with NAC, apocynin or YC-1, but was unaffected by their vehicles (Figure 5A). Western blot analysis showed that IH exposure for 14 days, but not for 7 days, increased the protein expression of HIF-1α in laryngeal tissues (Figure 5B and Supplementary Figure S1). This increased in the laryngeal expression of HIF-1α was completely prevented by daily treatment with NAC, but not its vehicle (Figure 5B and Supplementary Figure S1). Additionally, the expression of p47^phox^ in the membrane fraction, but not in the cytosol fraction, was higher in laryngeal tissues from 14 days IH rats than in RA rats. Administration of YC-1, but not its vehicle, totally inhibited the effect of IH on the expression of membrane p47^phox^ (Figure 6).

### Effectiveness and Selectivity for the Pharmacological Agents

In this study, the effectiveness of NAC and apocynin was evaluated by their total blockade of laryngeal lipid peroxidation induced by IH exposure for 14 days (Figure 5A); this is because activation of NADPH oxidase is known to be a major source of IH-induced ROS generation (Yang et al., 2016). The effectiveness of inhibitory effect of YC-1 was evaluated by laryngeal protein level of HIF-1α. Daily administration of YC-1 nearly abolished the elevation of HIF-1α expression in larynx induced by IH (Figure 5B and Supplementary Figure S1). The selectivity of those agents was illustrated by the persistence of apneic reflex evoked by laryngeal provocation of capsaicin, PBG or α,β-meATP in RA rats-treated pharmacological agents (Table 1).

**TABLE 1:** Average apneic responses to laryngeal chemical stimulants in various study groups.

**Groups**	**RA (*n* = 10)**	**NAC + RA (*n* = 5)**	**Apocynin + RA (*n* = 5)**	**YC-1 + RA (*n* = 5)**
Apneic reflex responses to laryngeal stimulants: apneic duration/baseline *T*_E_ (%)				
Capsaicin	277.3 ± 13.3	255.8 ± 36.0	265.4 ± 43.9	260.3 ± 42.0
PBG	241.1 ± 22.6	270.2 ± 23.2	255.3 ± 11.3	245.8 ± 12.5
α,β-meATP	236.5 ± 18.6	237.7 ± 24.7	254.4 ± 18.4	248.0 ± 11.0

*Rats were exposed to 14-days room air (RA). Apneic reflex responses to capsaicin, phenylbiguanide (PBG), and α,β-methylene-ATP (α,β-meATP) were measured in RA rats and in RA rats treated with NAC (NAC + RA), apocynin (Apocynin + RA) or YC-1 (YC-1 + RA). Values are presented as the means ± SE. See legends in Figures 2, 4 for details. No significant differences were detected among RA rats with or without treatment with various pharmacological administrations in response to the same stimulant.*

## Discussion

Results from the first part of this study demonstrate that IH exposure for 14 days, but not for 7 days, enhanced apneic responses to laryngeal chemical stimulants as compared with those of RA exposure. The apneic responses to these laryngeal chemical provocations were completely blocked by the perineural capsaicin treatment of SLNs or by the sectioning of the SLNs, suggesting that the reflex was mediated through capsaicin-sensitive SLNs. We also believed that perineural capsaicin treatment selectively blocks the capsaicin-sensitive SLNs because of the persistence of apneic response induced by laryngeal mechanical stimulation. Thus, 14 days of IH exposure may sensitize these afferents leading to elicitation of the augmented apneic responses to stimulants.

Capsaicin-sensitive SLNs are the majority of sensory afferents innervating the larynx and are sensitive to a variety of chemical stimulants (Tsai et al., 2009; Liu et al., 2013). In the present study, we used the three different stimulants: capsaicin, PBG, and α,β-meATP to stimulate these afferents by activating TRPV1 (Ruan et al., 2014), 5-HT3 (Hong et al., 1998), and P2X receptors (Ruan et al., 2014), respectively, located at the afferent terminals. Since the apneic responses to three chemical stimulants were all augmented, the IH-induced sensitization of these laryngeal afferents may not be a result of modulation of a single receptor type, but may be due to a non-specific effect on the increased sensitivity of these afferents. These findings are consistent with observations from our previous study (Yang et al., 2016). In that study (Yang et al., 2016), 14 days IH also markedly potentiated the sensitivity of capsaicin-sensitive vagal afferents in the lower airways to these chemical stimulants leading to lower airway hyperreactivity, a manifestation of asthma. Clinical studies have reported that patients with OSA are associated with laryngeal hyperreactivity (Nandwani et al., 1998) and asthma (Teodorescu et al., 2012). It appears that IH detrimentally impact both upper and lower airways in a similar fashion regarding their reactivity to stimuli.

Results from the second part of this study demonstrate that the 14 days IH-induced augmented apneic responses were significantly suppressed by daily treatment with NAC (a ROS scavenger), apocynin (an inhibitor of NADPH oxidase) or YC-1 (an inhibitor of HIF-1α), indicating the involvements of ROS, NADPH oxidase, and HIF-1 activation. Moreover, the apneic responses to three laryngeal stimulants were unaffected by daily treatment with NAC, apocynin or YC-1 in RA rats. Thus, the suppressive effects of these agents are unlikely resulting from impairing sensory neuron function because they did not affect the apneic responses to three stimulants in RA rats. Indeed, our biochemical analysis revealed that levels of lipid peroxidation, p47^phox^ in the membrane fraction, and HIF-1α expression in laryngeal tissues were all elevated following 14 days IH. The fact that these drugs reduced the augmented apneic responses to a similar level, suggests that ROS, NADPH oxidase, and HIF-1 activation are all essential regulators participating in the development of IH-induced LAH.

The involvements of ROS and NADPH oxidase in the augmented response are not surprising. ROS are known to be able to sensitize various types of capsaicin-sensitive vagal afferents (Lee and Yu, 2014). For example, in an animal model of gastroesophageal reflux disease, excessive ROS in the larynx may sensitize capsaicin-sensitive SLNs and induce LAH (Tsai et al., 2006; 2009). Additionally, we recently found (Yang et al., 2016) that ROS are essential for the sensitization of capsaicin-sensitive vagal afferents and the development of lower airway hyperreactivity induced by 14 days IH. The activation of NADPH oxidase is known to be a major source of ROS in various tissues in response to IH (Peng et al., 2009; Williams et al., 2015; Zhang et al., 2016; Liu et al., 2019). Indeed, the increased levels of lipid peroxidation in laryngeal tissues observed in this study were totally inhibited by apocynin, suggesting that ROS may be derived from the activation of NADPH oxidase. The NADPH oxidase-derived ROS has also been shown to contribute to the development of lower airway hyperreactivity induced by 14 days IH (Yang et al., 2016).

The mechanisms by which NADPH oxidase-derived ROS and HIF-1 activation are associated with IH-induced LAH remain unclear. In this study, rats exposed to 7 days exhibited increased laryngeal level of lipid peroxidation. At this time, the augmented apneic response to laryngeal stimulants was not found. These findings suggest 7 days IH-induced ROS generation may be not enough to induce LAH. Indeed, HIF-1α is believed to be responsible for mediating sustained ROS generation (Peng et al., 2006; Belaidi et al., 2016; Semenza and Prabhakar, 2018). Several studies from experimental animals (Peng et al., 2006; Semenza and Prabhakar, 2007) and cultured cells (Yuan et al., 2008) have reported that IH-induced ROS generation can increase HIF-1α expression. We also found that 7 days IH mildly, but significantly increased laryngeal lipid peroxidation, whereas it did not affect HIF-1α protein expression. In 14 days IH rats, laryngeal levels of lipid peroxidation and HIF-1α expression were markedly elevated. The former was reduced by YC-1, whereas the latter was inhibited by NAC. Collectively, these observations support the notion that IH may initially increase ROS generation resulting in HIF-1 activation, which in turn, promotes ROS generation in laryngeal tissues. In addition to the increased lipid peroxidation, 14 days IH rats exhibited elevated activation of NADPH oxidase in laryngeal tissues and this response was totally suppressed by treatment with YC-1, suggesting that HIF-1α mediates activation of NADPH oxidase. The interplay between increased HIF-1α expression and activation of NADPH oxidase has also been reported previously (Yuan et al., 2008, 2011). Thus, upregulation of HIF-1α expression may provide an important link between the initial increase in ROS and subsequent a more persistent increase in ROS via activation of NADPH oxidase in our IH model. Once ROS are overproduced and sustained a high level in laryngeal tissues, this result might sensitize capsaicin-sensitive SLNs and development of LAH induced by IH.

In this study, 14 days IH enhanced apneic response to laryngeal chemical stimulants; this apnea was completely abolished by perineural capsaicin treatment of SLNs, suggesting that capsaicin-sensitive SLNs may be involved. Due to the difficulty in performing the teased-fiber single-unit recording technique of SLNs, whether IH has any sensitizing effect on the afferents is still unknown. This notion is supported by a fact that ROS, a key mediator in the present study, has been reported to sensitize capsaicin-sensitive SLNs (Tsai et al., 2009), and then leads to exaggerated apneic reflex to laryngeal stimulants. In addition to sensitization of laryngeal afferents, enhanced apneic reflex could be attributable to changes in upstream inflammatory mediators or in downstream central pathways under IH exposure. IH is a systemic perturbation that can affect ROS generation and oxidative stress pathways across many tissues, including within vagal neurons themselves. A previous study (Andre et al., 2008) has shown that ROS can increases the excitability of guinea pig jugular ganglia neurons. In addition, IH has been shown to elevate baseline neuron activity in nucleus tractus solitaries (Kline et al., 2007), a brainstem respiratory center, which might then lead to the augmented reflexive behaviors. Thus, a possibility that potentiating effect of IH on apneic reflex is due to its action on vagal neurons and/or the central nervous system should be considered.

Several clinical studies have demonstrated that patients with OSA are associated with various types of LAH such as chronic cough (Sundar and Daly, 2011; Chan et al., 2015; Gouveia et al., 2019). Sensitization of laryngeal afferents has been implicated in the pathogenesis of chronic cough (Novakovic and MacKay, 2015). Patients with OSA also exhibit increased circulating levels of oxidative stress (Chen et al., 2019) and HIF-1α protein (Lu et al., 2017). These investigators found that the patients receiving therapeutic nasal continuous positive airway pressure for preventing repetitive episodes of upper airway collapse during sleep; this therapy is also effective in alleviating cough (Mutoh et al., 2000; Sundar and Daly, 2011; Novakovic and MacKay, 2015) and reducing oxidative stress (Chen et al., 2019) and HIF-1α level (Lu et al., 2017). These observations highlight the link among excessive ROS, HIF-1 activation, and LAH in patients with OSA. In this study, reflex apneic responses to laryngeal stimulants were measured to reflect capsaicin-sensitive SLNs-mediated LAH. Herein, we established an animal model to support the contributions of ROS, HIF-1α, and activation of NADPH oxidase to OSA-related LAH.

In summary, the results from the present study suggest that 14 days IH may sensitize capsaicin-sensitive SLNs. This sensitizing effect, at least in part, results in the non-specific increase in the excitability of the capsaicin-sensitive SLNs, which may augment apneic responses to laryngeal provocations with chemical stimulants. This sensitizing effect of IH may due to IH exposure initially cause an increase in ROS, which in turn activates HIF-1 in laryngeal tissues. HIF-1 activation then promotes ROS generated by activation of NADPH oxidase. Our findings provide novel information for understanding the pathophysiological mechanisms of OSA-related LAH and its potential therapy.

## Data Availability Statement

All datasets presented in this study are included in the article/ Supplementary Material.

## Ethics Statement

The animal study was reviewed and approved by the Institutional Animal Care and Use Committee of Tzu Chi University.

## Author Contributions

YL and CL contributed to the concept and design of the research, wrote, and finished the manuscript. Y-JS and P-HO performed the experiments. YL, Y-JS, P-HO, and CL analyzed and interpreted the data. All authors contributed to the article and approved the submitted version.

## Conflict of Interest

The authors declare that the research was conducted in the absence of any commercial or financial relationships that could be construed as a potential conflict of interest.

## Supplementary Material

Supplementary Figure 1 The whole blotting membrane of HIF-1α in the laryngeal tissues of RA, IH7, IH14, Vehicle + IH14, NAC + IH14, and YC-1 + IH14 rats were analyzed to assess HIF-1 activation. See the legend in Figure 5B for further explanation.
