# Supplementary figures and images for: HIF-1α-Mediated, NADPH Oxidase-Derived ROS Contributes to Laryngeal Airway Hyperreactivity Induced by Intermittent Hypoxia in Rats
Source: Front Physiol. 2020 Oct 7;11:575260. doi: 10.3389/fphys.2020.575260 (PMC7575773; doi:10.3389/fphys.2020.575260)

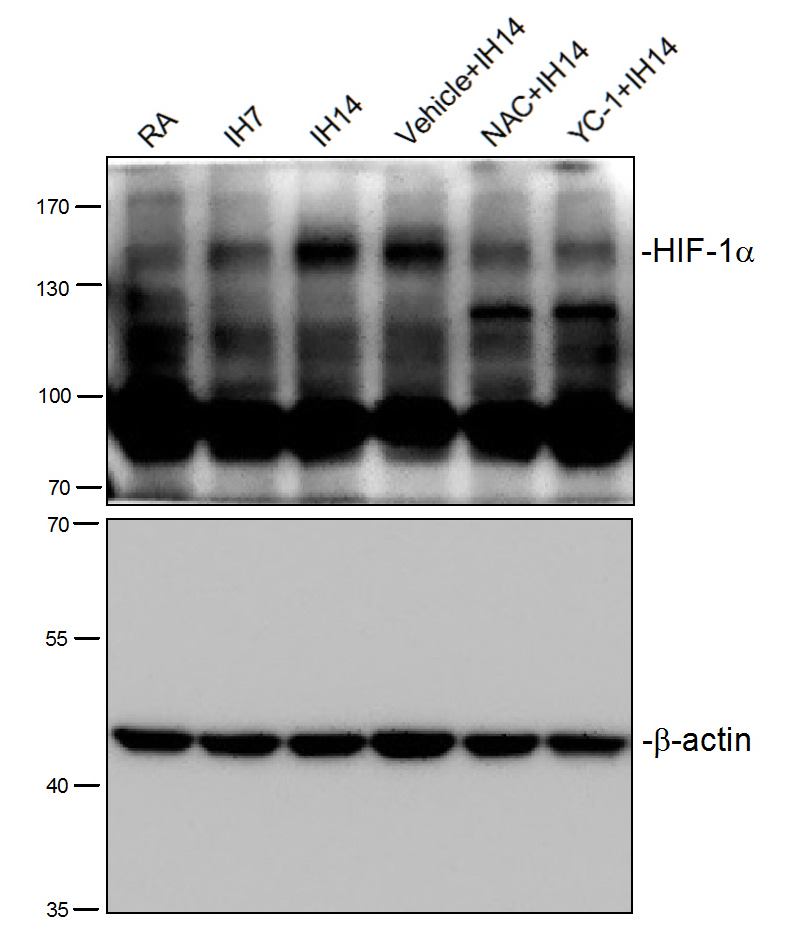

Supplement: Supplementary Figure 1 — The whole blotting membrane of HIF-1α in the laryngeal tissues of RA, IH7, IH14, Vehicle + IH14, NAC + IH14, and YC-1 + IH14 rats were analyzed to assess HIF-1 activation. See the legend in Figure 5B for further explanation. [file Image_1.jpg]
